# A Question of Ethics

**Published:** 1881-06

**Authors:** 


### A QUESTION OF ETHICS.

We clip the following from the editorial columns of the *Michigan Medical News*, and commend the sentiment therein contained:

A question of ethics has recently agitated the English profession, nor has the question been without interest in this country. The Earl of Beaconsfield, as everybody knows, was taken sick a couple of months ago with what proved to be his last illness. He sent for Dr. Kidd, the gentleman who attended him during his memorable sickness in Berlin several years ago. Dr. Kidd, as everybody also knows, thanks to the Atlantic cable and the wonderful network of wires connecting with either end, is a homœopath, at least the homœopaths claim him, and the "regulars" do not dispute the claim. But Beaconsfield was really sick this time and something more than a relinquishment of wine and generous living was necessary. The Empress Victoria, between whom and her late premier there was a strong bond of friendship, becoming alarmed for his safety, requested one of her numerous family physicians, Sir Wm. Jenner, to call round and see him. Sir William, however, politely refused to meet Dr. Kidd. "I must decline the request," said he, "for between us no agreement is possible and compromise with me is impossible." Sir William Gull, the gentleman whose ethical irregularities in connection with the recent Guy's hospital affair brought down on him the merited rebuke of the English profession, was then requested to see the Earl with Dr. Kidd. But he too, although he recently defended conduct universally condemned, refused to meet the homœopath even when told the homœopathic treatment was no longer being followed in the case. The next person requested was Dr. Quain. Dr. Quain after much deliberation and some consultation with his friends, and after the assurance from Dr. Kidd that he had abandoned homœopathy in his treatment of the case, concluded to meet the latter at the Earl's bedside. Beaconsfield hence died *secundem artem.*

Thus the matter was disposed of as far as the immediate necessities of the case were concerned, but Dr. Quain's conduct in consenting to meet the quasi homœopath was but the beginning of an acrimonious discussion on the ethical propriety of his having done so, a discussion which superadded to Sir William Jenner's and Sir William Gull's conduct is scarcely calculated to intensify the love of the people for regular medicine, and correspondingly gives homœopathy a boost among all who sherish the Englishman's proverbial love of fair play.

In our opinion the conduct of those who refused to meet Dr. Kidd was not justifiable under the circumstances. This gentleman, although popularly known as a homœopath, himself repudiates the name and certainly a perusal of his little book "Law in Therapeutics" will exhonorate him from the charge of dogmatism. It is true he treats most of his cases on the substitutive plan (the law of similars), but by no means believes in the universality of the law. He does precisely in this regard what every intelligent physician does, he does what he honestly believes is the best thing for his patient. We believe he errs in his judgment regarding the extent to which he finds the "substitutive" plan (this is a better name than "homœopathic") applicable, but this is his privilege, and who are we that we should sit in judgment in an affair of conscience? In the case in which the "regular" medical gentlemen were requested to meet him in counsel he was not prescribing after the substitutive plan, and as he is an educated gentleman, we regard Dr. Quain's action in meeting him as not only ethically proper, but, higher than ethics, humane. It is a narrow interpetation of the code which runs counter to humanity. Had Dr. Kidd been treating the case homœopathically and contrary to Sir William Jenner's views as to the correctness of this plan, his "no agreement possible, and compromise with me impossible," would have been the only course possible, but under the circumstances we cannot endorse the rhapsodies into which some of our exchanges have worked themselves on this utterance.

If homœopathy is to be fought it must be through showing the people that the regular profession regard humanity as superior to ethics, and by their not regarding it as a crime, for an educated gentleman to believe that it is possible to abort an idiopathic affection by the substitution of an artificial. *The time is coming, we believe, when homœopathy will be fought at the bedside, where alone it is possible to expose the fallacy of its acceptance as a universal law of cure.*—(Italic is our own—Ed. Atlanta Medical and Surgical Journal). The plan of heaping abuse on the heads of its practitioners has, at all events, proven a failure, and the time is ripe for some different mode of attack.

